# International Perspective on the Pursuit of Quality in Cancer Care: Global Application of QOPI and QOPI Certification

**DOI:** 10.1200/GO.20.00048

**Published:** 2020-05-06

**Authors:** Douglas W. Blayney, Nafisa Albdelhafeez, Abdul Rahman Jazieh, Carlos Frederico Pinto, Adrian Udrea, Alex Roach, Devika Das, Stephen Grubbs, John Hamm, Mohammad Jahanzeb, Arif H. Kamal, Ronan J. Kelly, S. Eric Martin, Deirdre O'Mahony, Walter Birch, Ronda Bowman, Stéphanie T. S. Crist, Amy Evers, Terry Gilmore, Meredith Klein, Robert Siegel

**Affiliations:** ^1^ Stanford Cancer Institute, Stanford University, Stanford, CA; ^2^ Ministry of National Guard and Health Affairs, Saudi Arabia; ^3^ Instituto de Oncologia do Vale, Sao Paolo, Brazil; ^4^ Medisprof Srl, Cluj, Romania; ^5^ American Society of Clinical Oncology, Alexandria, VA; ^6^ Hematology Oncology at The Kirklin Clinic of UAB Hospital, Birmingham, AL; ^7^ Norton Health Care Medical Oncology and Hematology, Louisville, KY; ^8^ Florida Precision Oncology, Boca Raton, FL; ^9^ Duke University School of Medicine, Durham, NC; ^10^ Baylor University Medical Center, Dallas, TX; ^11^ Medical Oncology Hematology Consultants, Newark, DE; ^12^ Cork University Hospital, Ireland; ^13^ Bon Secours St Francis Health System, Greenville, SC

## INTRODUCTION

In 2006, ASCO launched the Quality Oncology Practice Initiative (QOPI)^1^ for its US members to create a standardized mechanism for multidisciplinary oncology practices to measure quality and benchmark performance to inform continuous quality improvement. Since its inception, 6,806 practices have participated, with 490,294 records (as of May 2019) included in the registry.

In 2015, in response to demand from practices outside the United States, ASCO expanded QOPI participation to international partners, growing ASCO’s global quality impact. As of December 2019, the legal and regulatory framework exists for practices to participate in QOPI that have at least one ASCO member from Argentina, Australia, Brazil, India, Mexico, New Zealand, Pakistan, the Philippines, Saudi Arabia, and all countries within the European Union. For members seeking additional recognition for structure and processes for delivery of care that reflect the highest cancer care delivery standards in the field, international practices may seek QOPI Certification. Importantly, practices may elect to participate in QOPI without applying for QOPI Certification, because QOPI Certification may not be available to all countries where QOPI is available.

ASCO aims to expand the number of QOPI participants and QOPI-Certified practices worldwide in alignment with its goal to increase and exchange demonstrable and reproducible quality of cancer care delivery on a global scale. To assist with realizing this goal, the ASCO International Quality Task Force wrote this article as a guide for practices outside of the United States to participate in QOPI and pursue QOPI Certification. Highlights and experiences from the first practices to achieve QOPI Certification in Brazil, Romania, and Saudi Arabia are also included to provide a palpable view from colleagues internationally.

### International Participation in QOPI Certification

The QOPI Certification Program (QCP) provides a 3-year certification to oncology practices that recognizes exemplary commitment to safety and quality in oncology patient care. Presently, QCP is available to practices in all countries within the European Union, Brazil, Mexico, New Zealand, Pakistan, Romania, and Saudi Arabia. QCP operates as an educational partnership and sets a standard whereby practices that meet the high quality and safety standards are recognized. In 2016, the first practice outside of the United States achieved QOPI Certification. Since then, 14 practices from five countries (Brazil, Greece, Romania, Saudi Arabia, and Spain) have achieved QOPI Certification, creating a network of more than 300 certified practices worldwide. QOPI Certification is recognized globally as a leading program in collaborating with practices to implement sustainable quality improvements in oncology care, safeguarding both practices and patients.

The ASCO International Quality Task Force has identified six key steps to pursue QOPI Certification. These are highlighted below.

#### *1. Assess QOPI availability and eligibility.*

Determine if QOPI is available in your country (QOPI is currently available to all countries within the European Union, Argentina, Australia, Brazil, India, Mexico, New Zealand, Pakistan, the Philippines, and Saudi Arabia). If you are interested in bringing QOPI to your country, please complete our International Interest Survey.^7^ Practices should prepare to participate in QOPI by designating a QOPI Champion (ie, a team lead) to coordinate participation and manage deadlines.

ASCO recommends reviewing the QOPI registration tutorial video^8^ available at practice.asco.org^1^ before registering your practice. Registration is available at myQOPI.asco.org. After completing registration, practices will gain access to several tools to prepare your practice for participation in QOPI, such as Chart Selection Criteria and QOPI Abstractor Tips.

ASCO has produced country-specific QOPI and QCP agreements that can vary by country or region. Practices should consult with their legal team to review and sign both agreements applicable to their country. For QOPI Certification, international practices should review the pricing for countries outside of the United States.

#### *2. Participate in QOPI.*

QOPI is a quality assessment and self-reporting program. Practices abstract patient data into QOPI’s protected health information–secure system. Before data abstraction, each QOPI abstractor should view the QOPI Abstraction Training webinar available on the QOPI Dashboard^5^ or participate in a virtual training provided by QOPI staff. Once data have been entered, practices gain access to a robust set of reports based on the quality measures selected. These reports provide valuable individual performance scores by practice, site, and provider, as well as benchmarked scores aggregated from all participating similar practices. The QOPI measures, platform, and training materials are only available in English. Patient consents are available in Spanish, Portuguese, Hindi, and Urdu for countries that require patient consent for data submission. Practices interested in QOPI Certification should select only the QCP Track—comprised of 26 measures—during registration^2^. Practices participating in the QCP Track may also select additional modules, but this is not required, nor will it count toward their final overall quality score for certification.

To be eligible for certification, practices must achieve a 75%-or-greater overall quality score (valid for 1 year) on their final report and meet a minimum number of unique charts. The overall quality score determines eligibility and provides an opportunity for the practice to evaluate QCP survey readiness. Participation in QCP is optional. QOPI-measures assessment is an educational and informative tool for continual quality improvement. Practices can participate in the QCP Track in each QOPI round as often as they wish without penalty to meet the overall quality score.

QOPI participating practices also have access to the QOPI Help Desk. The QOPI Help Desk team supports practices with questions from registration to minimum target and unique charts needed for abstraction. Practices can contact the QOPI Help Desk via e-mail at QOPI@asco.org.

#### *3. Apply for QOPI Certification.*

Practices must achieve 75% or greater on the overall quality score in the QCP Track and abstract the minimum number of unique charts to apply for certification. Before applying, practices are required to participate in a web conference with ASCO staff to review the basics of the certification process and review in detail the QCP standards^3^. It is important to note the QCP standards examine the quality of cancer care in addition to the QCP Track measures by requiring practices to submit policies, procedures, guidelines, certifications, and/or staff rosters, patient education, and health care provider education related to select QCP standards. ASCO is available to provide this web conference in English and with a Portuguese translator upon request. QCP standards are currently available in English, Portuguese, and Spanish. For practices seeking to participate from Spain, the Foundation for Excellence and Quality in Oncology^6^ currently collaborates with QCP to assist practices in Spain with pursuing certification. If your practice is located in Spain, please contact the Foundation at fundacioneco@fundacioneco.es for additional assistance.

The QOPI Certification application is available through the QOPI portal for eligible practices^4^. Once all the application steps are complete, a practice is considered ready for its on-site survey. The application consists of six steps:

1. Eligibility
2. Agreements
3. QCP questionnaire
4. Payment
5. Presurvey documents
6. On-site survey availability.

Once all the application steps are complete, a practice is considered ready for its on-site survey.

ASCO will collaborate with international practices to prepare a custom QCP participation agreement and an invoice for payment via wire transfer. All submitted QCP documents (ie, policy documents and compliance documentation) are required to be submitted in English.

The QCP Help Desk is available to assist with all certification questions about the QCP application, on-site survey processes, and standards compliance. Practices can contact the QCP Help Desk via e-mail at QOPICertification@asco.org.

#### *4. Prepare for and participate in an on-site survey.*

All international surveys must be scheduled at least 3 months in advance (or longer if needed to accommodate visa applications). This survey serves as a collaborative educational opportunity for practices to meet standards. Practices will be notified of their on-site surveyor(s), confirm no conflicts of interest exist, and receive a date for the surveyor’s visit. All surveyor(s) are experienced QOPI Certification staff. For first-time on-site surveys in new countries, an ASCO physician member from the International Quality Taskforce will accompany the surveyor to the practice. Practices that require a translator are required to reserve a translator whose primary role is to provide translation services during the survey. During the on-site survey, the practice’s compliance with the certification standards will be evaluated on the basis of policy and records review, staff interviews, and observation of practice procedures.

#### *5. Receive on-site survey report.*

After the survey, a Committee reviewer will assess the surveyor’s observations to determine whether the practice successfully met all Certification standards. After the on-site survey, practices receive their Certification Compliance Report (CCR), documenting their level of compliance with each standard. If a practice fully meets each standard, the practice will be certified. If not, practices will be advised of any deficiencies in their CCR and must submit a Compliance Action Plan within 10 days to address the deficiencies. The Compliance Action Plan describes actions the practice will take to fulfill unmet standards as outlined in the CCR. In some cases, a resurvey may be necessary. Once the compliance plan is accepted by QCP staff, the implementation must be completed within 90 days. After the Compliance Action Plan is implemented and documentation is submitted, a Committee reviewer will assess whether the practice successfully met the requirements to achieve certification.

#### *6. Receive certification decision.*

Once the practice is 100% compliant with all standards, it is awarded QOPI Certification for a 3-year term. Certified practices will receive a complimentary certification plaque and be celebrated on ASCO’s website. In addition, ASCO will collaborate with the practice to produce a media kit to celebrate the practice’s achievement, including templates for social media and a local press release.

### Supplemental Guides for QOPI and QOPI Certification

#### *1. Gantt chart for QOPI Certification.*

**FIG 1:**
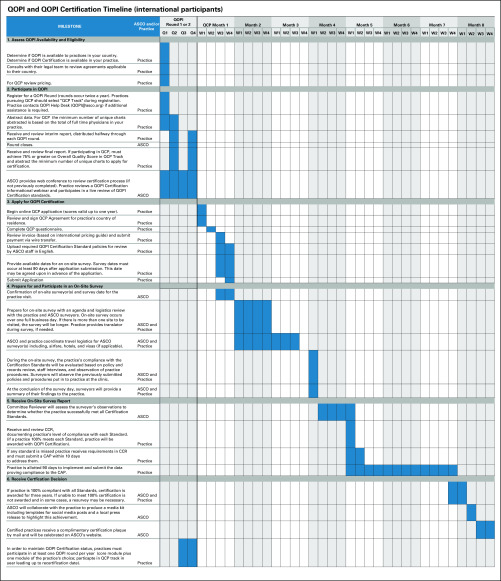
Process map for Quality Oncology Practice Initiative (QOPI) and QOPI Certification. The process map outlines key decision points made by both ASCO and the practice. CAP, compliance action plan; CCR, Certification Compliance Report; Q, quarter; QCP, QOPI Certification Program; W, week.

The Gantt chart (Fig 1) illustrates the timeline beginning with a practice’s participation in the QCP Track of one of the two QOPI rounds available each year. This timeline assumes QOPI and QCP are available in the country where the practice is located, the practice has the resources available to pursue QOPI Certification, and the practice has the support of its leadership and funding to pursue this initiative. We estimate achieving QOPI Certification will take most practices 9 to 12 months after meeting the benchmark to apply for certification.

#### *2. Process map for QOPI and QOPI Certification.*

**FIG 2:**
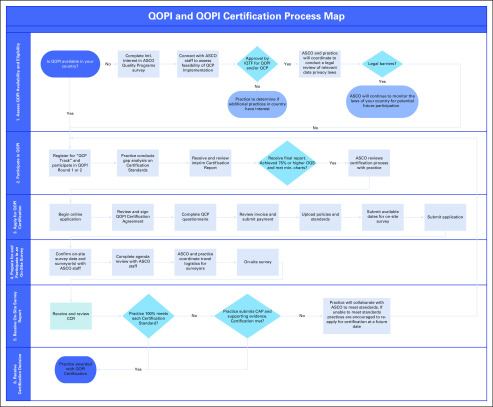
Quality Oncology Practice Initiative (QOPI) and QOPI Certification map. (*) In some cases, a resurvey will be necessary. A notification will be provided to the practice along with the Certification Compliance Report (CCR). ASCO will meet with practice to determine if a resurvey is recommended. CAP, compliance action plan; Intl., international; IQTF, International Quality Task Force; min., minimum; OQS, overall quality score; QCP, QOPI Certification Program.

The process map (Fig 2) outlines key decision points made by both ASCO and the practice during a practice’s pursuit to achieve QOPI Certification. The map also provides insight to the legal process for expanding QOPI availability to new countries. ASCO hopes to continue to expand to additional countries as resources allow.

## LESSONS LEARNED FROM INTERNATIONAL QOPI-CERTIFIED PRACTICES

We interviewed clinician and administration leaders from three international practices to better understand their motivations for participating in QOPI Certification, their approach to certification, and how their practice has been transformed in the process.

### QOPI and QOPI Certification: An International Perspective From Brazil

Dr Carlos Frederico Pinto, a medical oncologist and physician member of the Instituto de Oncologia Vale located in Sao Paolo, Brazil, led efforts to become the first QOPI-certified practice in Brazil in 2017. He describes the current state of oncology care in Brazil as “complex,” noting “the system is mixed between public (70%) and private (30%)” with “huge disparities between the two systems, limited access to breakthrough technology for the public system, and almost open access to the private sector.” Dr Pinto noted Brazil is currently “developing a quality culture” where the presence of quality programs has increased in the country in the past 10 years, but slowly. “Less than 10% of health care organizations in Brazil carry any kind of quality assurance certificate,” he says. ASCO hopes to expand the availability of its Quality Programs in Brazil by partnering with local hospital groups and professional societies.

QOPI Certification is awarded for a 3-year term with maintenance requirements occurring in 2 of the 3 years. In December 2019, Instituto de Oncologia Vale also became the first practice in Brazil to achieve recertification. Between the first certification in 2017 and applying to maintain its QOPI Certification status in 2019, Dr. Pinto noticed professional growth in the practice’s adherence to QCP standards. “One great achievement about QOPI (and all evolving systems) is that the bar is always moving up. From early days to our last survey, we evolved a lot with newly introduced standards at least once a year. It’s a great learning platform for improvement.” The maintenance requirement of participating in a Core module plus one module of their choosing allows practices to sustain their adherence to quality improvement measures.

ASCO’s strategic goals of driving high-quality care and increasing ASCO’s global engagement are evidenced by its growing collaboration with international practice participants in its Quality Programs. On ASCO’s pursuit of both goals Dr. Pinto comments, “I think ASCO is leading the way to improve cancer care worldwide. This is a challenge for us and for generations to come. Inequalities of care and access are the biggest challenges in health care.”

### QOPI and QOPI Certification: An International Perspective From Romania

ASCO’s QOPI Certification Program currently is the only oncology-specific international certification program available. Dr. Adrian Udrea, a medical oncologist at Medisprof located in Cluj, Romania, pursued QOPI Certification not long after opening the doors to Medisprof’s new hospital and was awarded QOPI Certification in 2018. Medisprof sought a mechanism to streamline its oncology procedures and policies in a new comprehensive cancer center and in its search for a certification program, Medisprof “considered ASCO QOPI Certification to be more adapted than other certification programs to medical oncology needs.”

Dr. Udrea highlighted some of the benefits of adapting to ASCO’s oncology-specific standards, citing that trainings dedicated to quality improvement and analysis for oncology professionals “are not required in our country but the administration know that quality of the services is linked to them.” Like many practices, Dr. Udrea notes that “at the beginning, it was hard to motivate the team to adapt to changes required by trainings and documentation of their work.” After achieving certification, however, Dr. Udrea and his team recommend international practices pursue this program because it is a great opportunity for professional development.

### QOPI and QOPI Certification: An International Perspective From Saudi Arabia

Dr. Nafisa Albdelhafeez and Dr. Abdul Rahman Jazieh realized the value of ASCO’s QOPI and Certification Programs during a visit from the immediate past chair of ASCO’s International Quality Task Force, Dr. Mohammad Jahanzeb, at the Ministry of National Guard – Health Affairs in Riyadh, Saudi Arabia. Dr. Jahanzeb provided a presentation highlighting the scope of QOPI and its new and expanding availability to countries outside of the United States in 2015. Both physicians realized the value of the program and decided to participate in the next QOPI round. “Our goal was to assess current status of our practice and to compare ourselves with the other practices. We were confident that our participation will enable us to get the maximum benefit from available resources and provide our patients with the best standard of care.”

Challenges in providing high-quality oncology care can vary by region. According to Dr. Albdelhafeez and Dr. Rahman Jazieh, some of the challenges experienced by their practice in participating in the program included developing standardized documentation and incorporating changes to their electronic health records system. Their advice to international practices looking to pursue Certification includes a commitment to “formulate a multidisciplinary team which meets regularly to make sure that all members work on harmony and understand the requirements.” In addition, they advise practices to “communicate with the ASCO-QOPI team whenever they have queries or unclear areas” because “communications can save time and effort.” ASCO encourages all practices to contact either the QOPI or QOPI Certification Help Desk to address their inquiries, because the programs are designed as an educational collaboration between your practice and ASCO.

In conclusion, QOPI and QOPI Certification provide benchmarking and guidelines for enhanced processes of care, and a modular map for the commitment to safety and quality that patients and providers must navigate to attain patient goals. Both programs provide opportunities for oncology communities globally to pursue continuous health care outcome improvements in a standardized manner.

ASCO hopes to continue to add to the increasing number of participants in QOPI and QOPI Certified practices and is working to provide QOPI availability to additional countries as resources allow. If you have any questions or are interested in participating in QOPI and/or the QOPI Certification Program, please contact globalquality@asco.org and complete our International Interest Survey.^7^
